# Robotic communication with ants

**DOI:** 10.1242/jeb.244106

**Published:** 2022-08-09

**Authors:** Nigel R. Franks, Jacob A. Podesta, Edward C. Jarvis, Alan Worley, Ana B. Sendova-Franks

**Affiliations:** 1 School of Biological Sciences, University of Bristol, 24 Tyndall Avenue, Bristol BS8 1TQ, UK; 2 Department of Biology, University of York, Wentworth Way, York YO10 5DD, UK

**Keywords:** Animal–robot interaction, Pheromones, Tandem running, Social behaviour, Learning, Orientation

## Abstract

We used a robotic gantry to test the hypothesis that tandem running in the ant *Temnothorax albipennis* can be successful in the absence of trail laying by the leader*.* Pheromone glands were placed on a pin attached to a gantry. This set-up substituted for the leader of a tandem run. Neither the pin nor the glands touched the substrate and thus the ant following the robot was tracking a plume of airborne pheromones. The robot led individual workers from their current nest to a potential new one. The robotic gantry was programmed to allow for human intervention along its path to permit the following ant to stop and survey its surroundings and then catch up with its mechanical leader. The gantry then automatically tracked the precise route taken by each ant from the new nest back to the old one. Ants led by the robot were significantly more successful at finding their way home than those we carried to the new nest that had no opportunity to learn landmarks. The robot was programmed to take either a straight or a sinusoidal path to the new nest. However, we found no significant difference in the abilities of ants that had been led on such direct or sinuous paths to find their way home. Here, the robot laid no trail but our findings suggest that, under such circumstances, the following ant may lay a trail to substitute for the missing one.

## Abstract (precis)

**Highlighted Article:** During tandem running, a leading ant teaches a follower the route to a resource. Key features of real tandem runs were successfully reproduced using a gantry as a robotic leader.

## INTRODUCTION

The study of behaviour and cognition is undergoing a relatively recent transformation through the use of robots that can interact with one or more animals and thus reveal how individuals signal to, or influence, one another (Krause et al., 2011; Mitri et al., 2013; Romano et al., 2019). Such robots are arguably a new form of the lures and models traditionally used in ethology (Tinbergen, 1951, pp. 27–46). In turn, the applied use of lures by humans to catch prey has a very long history indeed from decoy ducks (Ackerman et al., 2006; Chiarappa, 1997) to fishing with artificial flies, which was first recorded, at least in European history, by Claudius Aelianus, who wrote about Macedonians fly fishing 1800 years ago (Aelian, 1959, p. 205).

Behavioural investigations using robots can determine what is essential and what is peripheral to the way animals interact socially. Exemplary studies include the use of robotic fish to influence schooling behaviour (Li et al., 2021) and the employment of robotic cockroaches to understand aggregation formation (Halloy et al., 2007). Here, our aim was to use a robot better to understand communication in *Temnothorax* tandem-running ants. This is a particularly fascinating behaviour because tandem running was the first behaviour to be shown to meet all the criteria for teaching in animals (Franks and Richardson, 2006).

Caro and Hauser (1992) defined teaching through a set of criteria that can be paraphrased as follows. An individual is a teacher if (a) it modifies its behaviour in the presence of a naïve observer, (b) at some initial cost to itself, (c) in order to set an example, so that (d) the other individual can learn more quickly. In the first rigorous demonstration of teaching in non-human animals, Franks and Richardson (2006) showed that all of these criteria are met by ants tandem running to a potential new nest site.

Tandem running is a form of recruitment in which one ant leads a single follower to a site of importance such as a valuable food source or a new nest. The leader releases, into the air or onto its body, a short-range pheromone that elicits following by a single nest-mate. Tandem runs progress slowly because the follower frequently stops and turns around to learn landmarks so that it can find its own independent way home after it has visited the site to which it was led. The leader remains still during such interruptions until it is authorized, by contact from the follower, to continue. The contact takes the form of tapping by the follower with its antennae on the hind legs or gaster of the leader (Möglich et al., 1974).

During a potential emigration to a new nest site, ants use tandem running to build a quorum at the new site, after which ants are then carried, rather than being led, to the new nest. Ants led in tandem runs learn the route and can, in due course, lead tandem runs; carried ants are simply delivered to the new nest site and do not learn the route (Pratt et al., 2002). Hence, tandem-led ants and carried ants can be used to determine how much a tandem recruited ant has learnt in comparison with a naïve (carried) one.

An alternative strategy to tandem running is mass recruitment as used by ant species with large colonies. In this case, ants discovering a valuable resource lay and repeatedly reinforce a pheromone trail. This involves rapid positive feedback so that many ants can quickly utilize the resource. Mass recruitment, unlike tandem running, does not target specific individuals; rather, it is broadcast communication (Hölldobler and Wilson, 1990). Tandem running, in contrast, is found in ant species with small colony populations of workers (Hölldobler and Wilson, 1990). It is one-to-one tuition, and probably reduces the risk that individuals become lost. Such losses would be particularly costly for small colonies. In common with mass recruitment, tandem running in *Temnothorax* ants may actually involve some trail laying by the leading ant (Basari et al., 2014b), but such trails are neither slavishly followed nor reinforced; rather, they may serve as safety lines to help ensure that individuals do not become lost (Mcleman et al., 2002; Pratt et al., 2001). Behavioural studies have suggested which pheromone glands are used by *Temnothorax* ants for communication in different circumstances (Möglich, 1979; Möglich et al., 1974; Morgan, 2008; Provost et al., 1993; Qiu, 2021 preprint; Sasaki et al., 2020). However, we agree with Sasaki et al. (2014) that little is known of the pheromone chemistry of *Temnothorax*.

Here, we used a robotic leader, as a substitute for a real ant, to lead a live follower. Our goal was further to understand what elements are necessary and sufficient for successful teaching by tandem running in this fascinating form of communication. The benefit of using a robotic leader as a substitute for a real ant is that we can control the path the leader takes and, to some extent, the information available to a follower. Recently, Richardson et al. (2021) have shown that in tandem running by *Temnothorax* ants, good leaders are more important than good followers to the success of their endeavour. Using a robot as the leader of a tandem run should shed new light on what is most important in that role.

The robotic leader is a pin, carrying at its tip the pheromone glands used in tandem running and mounted on a computer-controlled gantry. The robotic leader may be programmed to take different forms of path to the new nest site. The gantry was equipped with a camera and a joystick so that the gantry operator could modulate the speed of the robotic leader to retain contact with the follower if the latter paused during the tandem run. The gantry camera was then used to auto-track and record the path of the follower back to the old nest once it had evaluated the new one.

We used the robotic leader in experiments to address the following questions. Are ants that are led by a robot better at finding their way home than ants that have been carried to the same site? Does the shape of the path, taken by the robotic leader between the old and the new nest sites influence the ability of follower ants to find their way home?

## MATERIALS AND METHODS

### The colonies

Ten colonies of *Temnothorax albipennis* Curtis 1854 used in these experiments were collected from south Dorset, UK, in January 2015. The colonies were kept in standard nests constructed from two glass slides (76×51 mm) with a cardboard gasket, 1.6 mm thick, separating them. A card cover was placed over the nests to keep a dark interior and these were housed in square Petri dishes (120×120 mm) that were individually labelled. The ants were fed weekly on honey solution and three to five freeze-killed, lab-reared *Drosophila*, and also provided with fresh water. The 10 experimental colonies were used and re-used sequentially to maximize recovery periods (Langridge et al., 2004). Any effect of the repeated use of the same colonies was taken into consideration within mixed-effects statistical models (see Statistical analysis).

### The arena

**Fig. 1.**
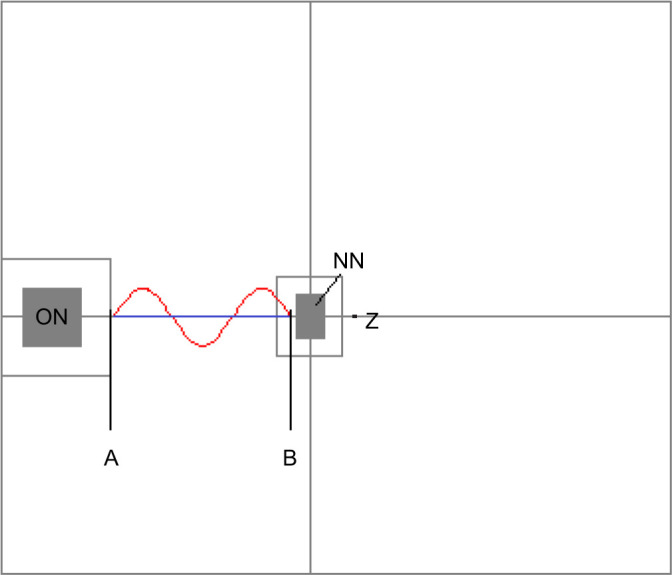
**The experimental arena was made of plexiglass with a matt white floor with dimensions 1134×980 mm (horizontal×vertical, not to scale).** ON: old nest; NN: new nest; A: 50 mm mark; B: 350 mm mark (a further mark was made at 360 mm, i.e. 10 mm beyond the 350 mm mark, where the new nest was placed); Z: the zeroing spot (a pencil mark behind the new nest, on which the camera was centred to provide a consistent reference for the recorded movement coordinates). Blue and red lines: the path of the robotic leader in the straight and sinusoidal treatments, respectively.

The old nest (the occupied nest from which ants were led or manually carried) was placed on the midline of the arena, but somewhat towards one end, with the nest entrance facing the centre of the arena (Fig. 1). A mark 50 mm in front of the old nest served as a reference point to start the gantry or stop the auto-tracking (Fig. 1, ‘A’). A second mark 300 mm further along the same line was used to indicate the start of auto-tracking on the return run (Fig. 1, ‘B’). At a further 10 mm forwards was the ‘360 mm’ mark. In treatments that required a second, empty nest (new nest), the entrance was placed on the 360 mm mark facing the old nest (Fig. 1, ‘NN’). The arena floor was white except for the marks, as described above, which were made with a pencil (such graphite marks do not interfere with the behaviour of the ants; N.R.F., personal observations). The arena was kept clear of dust and debris which could interfere with the auto-tracking procedure. During all experimentation, the laboratory windows were covered to control for variation in natural light at different times of day. Incidental landmarks (for example, the lab PC) were kept constant throughout. A Petri dish with a small window cut into it was placed over the old nest to ensure that a single exiting ant encountered the robotic leader (Fig. 1).

### The robotic leader

**Fig. 2.**
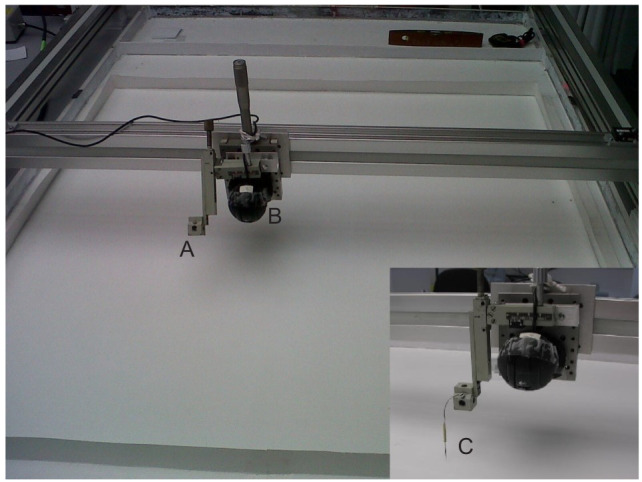
**The pin, C, was mounted at point A on an arm projecting from the gantry beam, and the camera was mounted at point B.** This set-up was used both to lead the ants to the new nest (not shown) and to track them on their return journey to the old nest (also not shown).

The robotic leader was mounted on the gantry as described by Basari et al. (2014a) with two additional features: auto-tracking and the ability to trace out a predetermined path. This robotic leader was used to lead ants on artificial tandem runs. The Dufour's and poison glands of individual ants were dissected out by the methods described by Möglich (1979). As the focal colony was introduced, two worker ants were removed from it and frozen for 10 min at −20°C (Liebherr Med Line freezer). Although some volatile compounds in the ant samples might be lost owing to such a freezing process (Chen, 2017), we nevertheless found that the robot-led ants performed significantly better than the carried ants. The Dufour's and poison glands were extracted from workers of the focal colony to prevent any confounding effects from colony-specific pheromones. The glands were then placed on the tip of an entomology pin fixed to a piece of wire. Only a single set of glands was used on the pin with each attempt, and the glands were not crushed. The wire holding the pin could be bent to adjust the position of the pin once mounted on the gantry (Fig. 2, ‘C’). The pin could then be raised or lowered by means of a screw-controlled rack-and-pinion mechanism (taken from a microscope stage) so that the pinhead was approximately 1 mm above the substrate. In this way, the pin tip was roughly at the height of a leader's gaster during a tandem run. Neither the pin nor the glands it was carrying were allowed to touch the substrate surface at any point. The robotic leader was then moved into position with the pin immediately in front of the exit from the Petri dish over the old nest. Once an ant was successfully led all the way to the new nest, all ants not involved in the run were removed from the arena and an intact Petri dish was placed over the old nest to prevent further ants from interfering with the auto-tracking.

### The program for the robotic leader

The method used artificially to lead the ants was as follows: once an ant had emerged and interacted with the pheromone-bearing pin, the gantry was moved forward slowly by manual control. If the ant kept following up to the 50 mm mark, the robotic leader program was started. The program allowed a straight path or a customisable sinusoidal path to be taken by the robotic leader. For the sinusoidal path, the following parameters could be set: amplitude (the maximum displacement from the central line), distance (the straight line distance between the start and finish points), half-cycles (the number of ‘peaks’ in the path) and speed (the initial speed of the robotic leader, modifiable with the joystick). The gantry could be manually controlled, by a joystick, before the sinusoidal movement had been started and after it was completed, allowing positioning of the pin and the initiation of runs. During the movement of the robotic leader along either type of path, the joystick could be used to adjust the speed of the robot from the pre-set value. Speeds plus or minus a factor of 10 of the inputted value could be reached, which, for practical purposes, allowed an almost complete stop. This ensured that the robotic leader could be made to wait when the follower paused and also to match the speed of the follower when moving. Such use of the joystick altered only the speed but did not cause deviations from the straight or sinusoidal path of the leader. This facilitated a closer resemblance to the bidirectional feedback seen in natural tandem runs. The program output included the speed and coordinates of the gantry every 0.1 s.

Once the robotic leader had reached the entrance of the new nest with an ant following, the pin was removed and the ant was led into the nest entrance by hand. The size and shape of the gantry did not allow the robotic leader to go to the entrance of the nest completely without interfering with the nest. Additionally, a natural tandem run would end with the leader entering the nest and the robotic leader could not. In order to prevent the follower from pausing with the robotic leader at the entrance instead of entering the new nest, when the robotic leader program finished (10 mm before the nest entrance), we quickly dismounted the pin (and its gland) and moved it gradually, allowing the follower to follow, so that the tip was at the entrance to the nest. We then removed the pin completely, to prevent the problem of the pin blocking or preventing the ant from entering the nest. At the time the ant entered the new nest to begin its assessment, a stopwatch was started. The times of all exits/entries to the new nest, starting/stopping of the auto-tracking program and arrivals at the old nest were recorded. At this point the robotic leader program was terminated and the auto-tracking component of the procedure was initiated.

### Auto-tracking

After an ant had been led to the new nest by the robotic leader and during the time it explored the new nest, the auto-tracking program was started. The camera was zeroed by centring it over a pencil mark behind the new nest for this purpose (Fig. 1, ‘Z’). This provided a consistent frame of reference for the coordinates saved in a text file by the auto-tracking program. Once zeroed, the camera was centred in front of the new nest entrance. If the ant failed to emerge within 20 min from the new nest entrance (Table S1), the run was aborted. If the ant did emerge, the auto-tracking program was initiated once the ant was more than 30 mm from the new nest (this distance was half of the *y*-component of the camera's field of view). This procedure was adopted because the auto-tracking software would detect the nest rather than the ant if the nest was visible. The software kept the camera centred upon the ant and saved the ant's coordinates and the time. The ant was deemed to have returned to the old nest when it was within 50 mm (Fig. 1, ‘A’) of the Petri dish covering the old nest. When the ant reached the old nest, the program was stopped and the final time was noted. If the ant did not return to the old nest within 30 min, it was deemed to have failed to find it and the trial was aborted. Given the arena size and the movement speed of the ants, 30 min was a reasonable time threshold.

The arena was then cleaned to remove any pheromone trails that may have been laid. The procedure for cleaning was in three steps. Firstly, the arena was wiped with soapy water on a paper towel. Next, a fresh paper towel was used to apply 70% ethanol solution to the arena surface. Finally, a towel dampened with purified water was used to wipe the arena to remove any potential residue.

### Treatments

#### Straight

Ants were led in a straight path to the new nest by the robot (amplitude=0 mm, distance=300 mm, half-cycles=0, speed=2 mm s^−1^, *n=*16).

#### Sinusoidal

Ants were led along a sinusoidal path by the robot (amplitude=50 mm, distance=300 mm, half-cycles=3, speed=2 mm s^−1^, *n=*15).

#### Cleaned

Ants were led along a straight path as above. However, the arena surface was cleaned whilst the ant explored the new nest (*n=*16). This controlled for any pheromones laid by the following ant or residue from the robotic leader. The cleaning procedure was the same as that used between nest trails.

#### Carried

Ants were removed from their nest with forceps and placed on the 360 mm mark (Fig. 1). Ants carried in this manner rarely leave the new nest when one is present (Pratt et al., 2002). Hence, we did not provide a new nest. Our goal was to determine how long it took such carried ants to return to the old nest site (*n=*15). To control for the possibility that scents from a real, occupied, old nest help returning ants find their home, we used a clean, unoccupied nest instead. This dummy old nest made the experiments involving carried ants comparable with the experiments involving robot-led ants where any nest scents were blocked by the intact Petri dish covering the old nest. The ant was considered to have returned when it reached the dummy nest in a similar way to the treatments in which a real, occupied nest was present. This allowed comparison between the success rate of individuals that had followed the robotic leader and ones that had received no such training.

#### Control

This was identical to the carried treatment, except that the robotic leader (including glands) was run beforehand along either a sinusoidal or a straight path but with no ants being led. Replicates were run at the average speed of the robotic leader for each of the sinusoidal and straight paths as calculated from the observed values during the straight (*n=*7) and sinusoidal (*n=*8) treatments (1.84 and 1.92 mm s^−1^, respectively). This was to control for the possibility that the robotic leader itself may have been dripping pheromone onto the arena floor and in effect laying a detectable trail that the ants could follow.

#### Filmed

This was identical to the carried treatment, but rather than being tracked with the gantry, the ants were filmed from above to control for any effect of the gantry presence on the return of ants. The position of the camera on the gantry (Fig. 2, ‘B’) was such that the gantry arm (Fig. 2, ‘A’) always cast a shadow on the same side and behind an ant returning to the old nest. In effect, this shadow inadvertently followed the returning ant. If the ant responded to this shadow, a directional bias towards the old nest might be expected in the treatments involving tracking by the gantry but not in the filmed treatment. We tested this with the null hypothesis that in the absence of any such bias, a returning ant would spend the same amount of time in each half of the arena: the half containing the old nest and the half behind the new nest. Hence, we recorded the time spent in each of the four arena quadrants (*n=*15). This was done by observing the time of the ant crossing the quadrant lines on an acetate sheet fixed to the computer screen during playback. The sheet was divided into four equal quadrants and centred at the point on which the ant was placed (the 360 mm mark). At the beginning of the recording, a stopwatch was started and the time recorded when the ant moved from one quadrant to another. This timeline was then used to calculate the time spent in each quadrant. The recording was stopped when the ant reached the old nest or after 30 min, whichever was sooner. This treatment was carried out in a smaller arena (600×900 mm) owing to constraints on the camera's field of view as it had to be mounted on a tripod and aimed vertically down to capture the focal ant's movement. Because variables such as return success were likely to be strongly affected by the arena size, only the quadrant data (percent time in each quadrant) could be compared with other treatments.

#### Simulated

This treatment provided a comparison between the behaviour of the ants and a correlated random walker with an unbiased initial orientation. A program recorded the path length of the simulated random walker and the number of times it crossed the sinusoidal path (*n=*15) or the straight path (*n=*15) that the robotic leader would have been programmed to take. The program sampled the turning angle of the walker randomly from a Gaussian distribution with a mean of 0 deg and an s.d. of 5 deg. These values were chosen based on our experience of the movements of *T. albipennis* ant workers (Mcleman et al., 2002; Pratt et al., 2001) and that of other researchers (Sasaki et al., 2020). The step length in the program was 2 mm because this approximates the length of a *T. albipennis* worker.

### Statistical analysis

We compared the return durations of ants under different treatments (except filmed and simulated) with survival analysis. This allowed the incorporation of the return durations of ants that did not return to the old nest within the permitted 30 min (Table 1) as censored data. In order to test for any effect of the random factor colony, for each analysis we fitted a Cox mixed-effects model and a Cox proportional hazards model (which included the same predictors except for the random factor colony) and then compared them. The effect of the random factor colony was not statistically significant in any of the analyses [straight, sinusoidal and cleaned: χ^2^=0.0058, d.f.=1, *P*=0.939; carried and control: χ^2^=0.1186, d.f.=1, *P*=0.731; robot-led ants (pooled straight, sinusoidal and cleaned) versus carried ants (pooled carried and control): χ^2^=0.0059, d.f.=1, *P*=0.939]. Indeed, in the first and third of the above analyses, the random effect of colony had a standard deviation of 0.020, that is exp(0.020)=1.020 or only 2% higher (lower) than the average likelihood of a return to the old nest for the ∼15% of ants 1 s.d. above (below) the average. However, in the second analysis, the random effect of colony had a standard deviation of 0.352, that is exp(0.352)=1.422 or 42% higher (lower) than the average likelihood of a return to the old nest for the ∼15% of ants 1 s.d. above (below) the average.

**Table 1.**
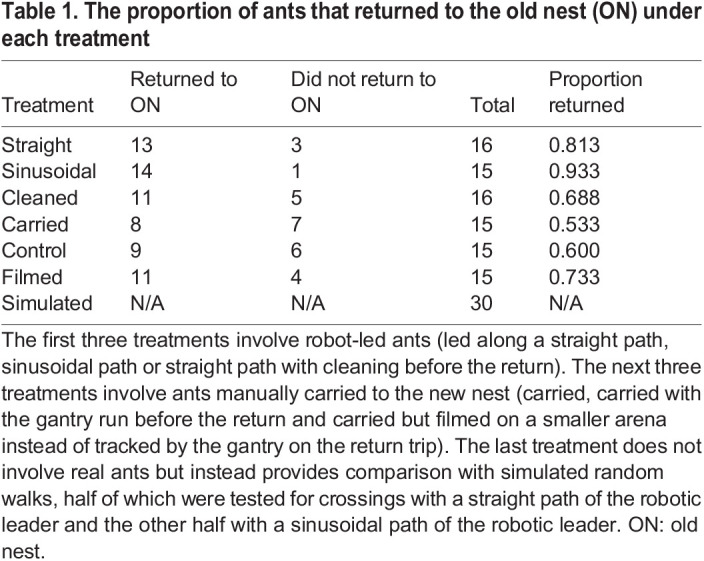
The proportion of ants that returned to the old nest (ON) under each treatment

For the analysis of the number of crossings between the return paths of focal ants and the robotic gantry, we used a Kruskal–Wallis test with Holm's test (Holm, 1979) for the *post hoc* comparisons between treatments. A general linear model and a Shapiro–Wilk test for normality of residuals were applied to test for any difference in the activity of carried ants when tracked by the gantry back to the old nest and when filmed with a camera. Finally, we used a Fisher's exact test to check for any differences in the odds of returning versus not returning to the new nest between followers of the robotic leader and ants carried manually to the new nest.

All statistical analyses and graphical representations of the data were carried out in R v. 4.1.0 (https://www.r-project.org/) with the additional packages coxme (https://CRAN.R-project.org/package=coxme) and survival (Therneau and Grambsch, 2000) for the survival analysis.

## RESULTS

### Home runs by ants led by the robot along paths of different shape

**Fig. 3.**
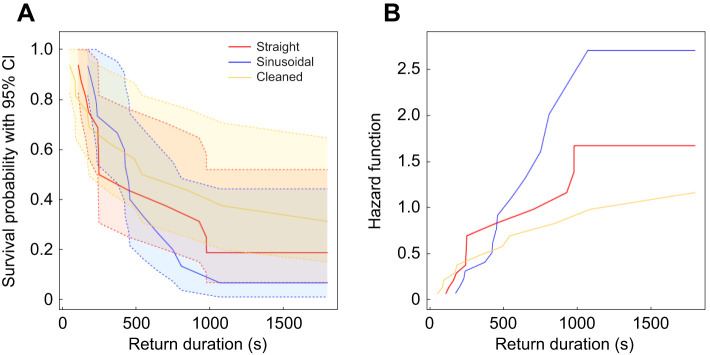
**Robot-led ants.** (A) Survival function and (B) hazard function for the return duration (s) of ants led by the robot on a straight path, on a sinusoidal path or on a straight path with the arena cleaned before they embark on their return journey.

We found no evidence that the shape of the path taken by the robotic leader between the old and the new nest influenced the ability of the robot-led ants to find their way home (straight versus sinusoidal; Fig. 3). The likelihood of a robot-led ant returning to the old nest increased on average by 24% when it was led on a sinusoidal than a straight path without cleaning. However, this difference was not statistically significant (Cox mixed-effects model: hazard rate=1.24, *z*=0.54, *P*=0.59).

### Home runs by robot-led ants with and without arena cleaning

We found no evidence that robot-led ants failed to find their way home in the absence of any trail laying by the robotic leader, or indeed themselves, on the outward journey (straight versus cleaned; Fig. 3). The likelihood of a robot-led ant returning to the old nest increased on average by 44% when it was led on a straight path without arena cleaning than on a straight path with arena cleaning before the return journey. However, this difference was not statistically significant (Cox mixed-effects model: hazard rate=1.44, *z*=0.89, *P*=0.37). Similarly, the likelihood of a robot-led ant returning to the old nest increased on average by 78% when it was led on a sinusoidal path than a straight path with cleaning (sinusoidal versus cleaned; Fig. 3). However, this difference was not statistically significant either (Cox mixed-effects model: hazard rate=1.78, *z*=1.40, *P*=0.16).

### Home runs by carried ants with and without a prior run of the robotic leader

**Fig. 4.**
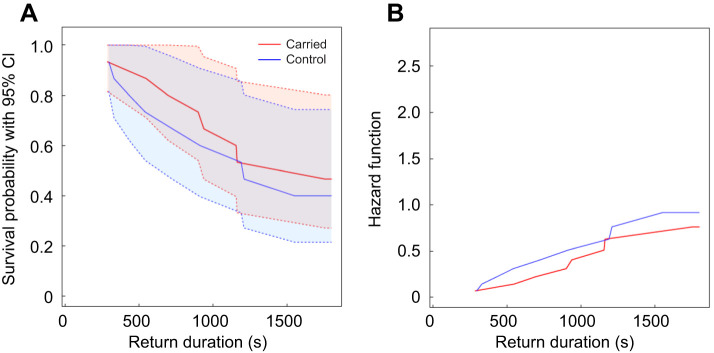
**Ants carried manually to the new nest.** (A) Survival function and (B) hazard function for the return duration (s) of ants manually placed at the new nest and tracked by the gantry on their return journey after the robotic leader was run (control) or not run (carried) on a straight or sinusoidal path without any followers.

**Fig. 5.**
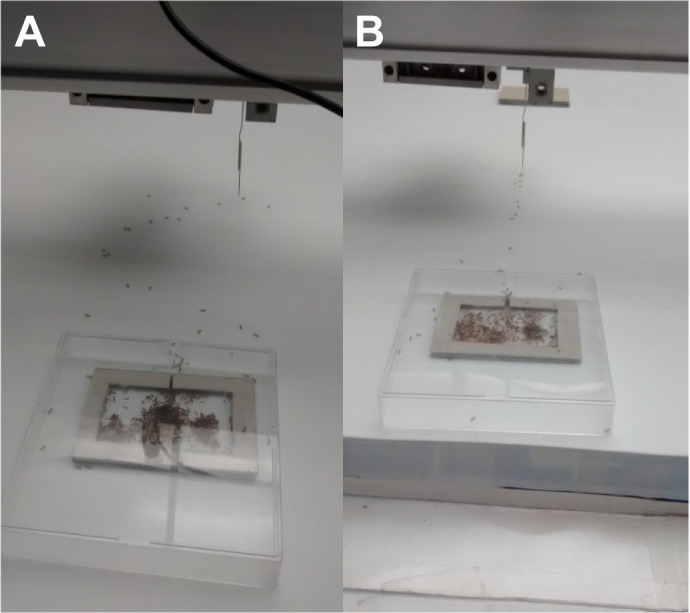
**Ants were occasionally observed clustered along the path taken by the gantry, perhaps responding to pheromone.** The gantry had been run on (A) a sinusoidal path and (B) a straight path. Our results indicate that this surface pheromone does not originate from the robotic leader itself.

We found no evidence that the run of the robotic leader prior to the return trip of carried ants influenced their return success (Fig. 4). The likelihood of a carried ant returning to the old nest increased on average by 36% when the robotic leader was run before its return journey. However, this difference was not statistically significant (Cox mixed-effects model: hazard rate=1.36, *z*=0.61, *P*=0.55). These results suggest strongly that the robotic leader was not leaking pheromone. The alternative is that the robot-led ants themselves were laying trails. They may do so on the arena surface to compensate for the lack of a safety trail laid by a tandem leader (Fig. 5).

### Crossings with the outward path of the robotic leader

**Fig. 6.**
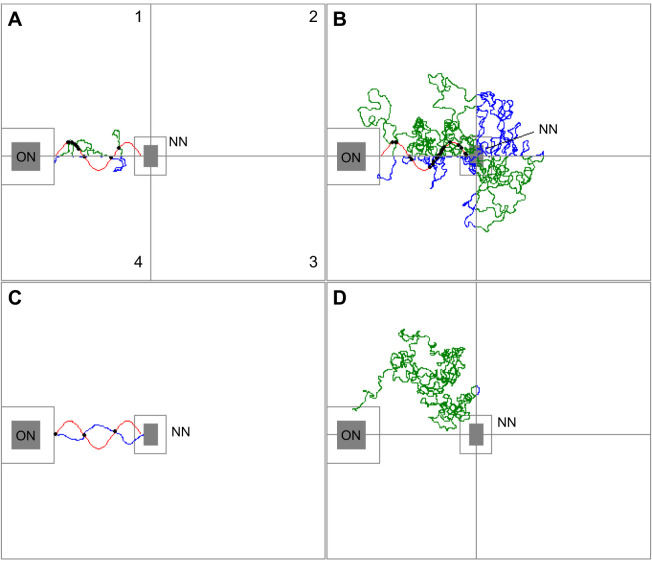
**Analysis of path crossing and activity in each arena quadrant.** The quadrants were labelled clockwise 1–4. The four examples given are: (A) a sinusoidal return: the ant was led by the robotic leader and allowed to return from the new nest; (B) a control carry: the ant was carried to the new nest but the robotic leader was run prior to its return without any followers; (C) a simulated run; and (D) a cleaned return: the ant was led by the robotic leader and the arena was cleaned of any pheromones before the ant began its return journey. ON: old nest (the area enclosed by the covering Petri dish; 120×120 mm); NN: new nest (the area of the vacant nest itself; 76×51 mm); area enclosed by the grey line around the filled grey rectangle of each nest: the nest for experimental purposes as this was the vicinity in which the ant could not normally be tracked; red line: the outward path of the robotic leader; green/blue line: the path taken by the returning ant in each quadrant; black dot: where a returning ant crossed the path of the outward robotic leader.

**Fig. 7.**
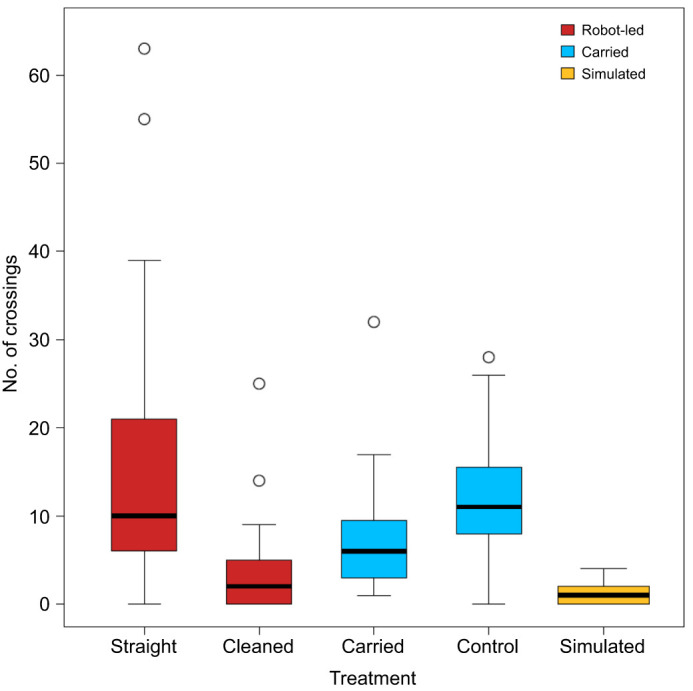
**Number of crossings.** The number of times the outward path of the robotic leader was crossed by ants returning to the old nest under different treatments. Red: robot-led ants; blue: carried ants; yellow: simulated ants (see Table 2). Box width is proportional to sample size.

The evidence for robot-led ants laying trails is strengthened further by the significant difference in the number of crossings of the robotic leader's path by returning ants under the different treatments (Kruskal–Wallis test: χ^2^=36.36, d.f.=4, *P*=2.45×10^−7^; Figs 6,7). In particular, the number of crossings was significantly smaller for robot-led ants when the arena was cleaned than when it was not cleaned (cleaned versus straight; Table 2). This suggests that a returning robot-led ant perceived the pheromone trail left either by the robotic leader or itself. A putative pheromone trail left by the robotic leader may also have been perceived by the returning carried ants for the following reason. The number of crossings they made after the robotic leader had been deployed was significantly greater than the number of crossings made by returning robot-led ants after the arena had been cleaned (control versus cleaned; Table 2). However, this difference might be attributable to their greater mobility and more erratic movements owing to the stress of being carried by forceps. Crucially, there was not a significant difference in the number of crossings when the robotic leader had or had not been run before a carried ant could return back to the old nest (control versus carried; Table 2). This means that any pheromone trail left on the arena is unlikely to have come from the robotic leader. In turn, this strongly suggests that a pheromone trail might have been left by the ant led by the robot. Finally, the number of crossings by robot-led ants returning to the old nest after the arena had been cleaned was not significantly different from that of simulated random walkers, even though such a difference existed between each of the other three treatments and the random walkers (Table 2).

**Table 2.**
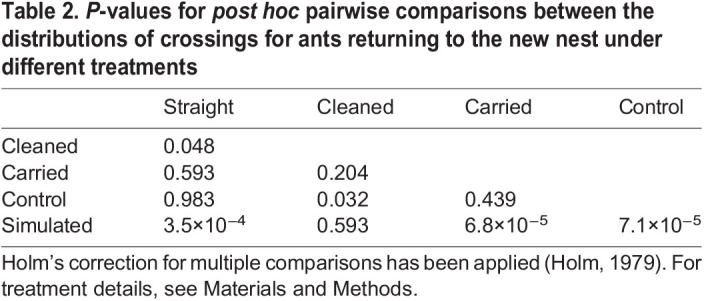
*P*-values for *post hoc* pairwise comparisons between the distributions of crossings for ants returning to the new nest under different treatments

### Home runs by robot-led ants compared with carried ants

**Fig. 8.**
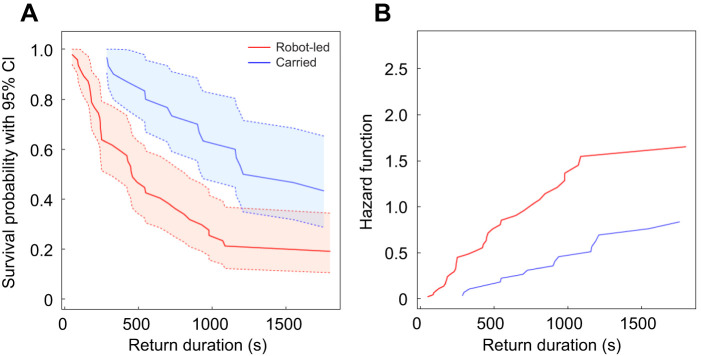
**Comparison between all robot-led ants and all carried ants.** (A) Survival function and (B) hazard function for the return duration (s) of all robot-led ants (on a straight path, on a sinusoidal path and on a straight path with the arena cleaned before their return journey) and of all carried ants (manually placed at the new nest and tracked by the gantry on their return after the robotic leader was or was not run on a straight or sinusoidal path without any followers; see also Table 1).

The ants led by the robotic leader were better at finding their way home than ants carried to the same site (Fig. 8, Table 1). We pooled the data for each of these two groups because we found no evidence of a difference in the return success between treatments within either the robot-led ants (Fig. 3) or the carried ants (Fig. 4). The likelihood of an ant returning to the old nest increased more than two-fold on average when it was led by the robotic leader than when it was carried manually to the new nest. This difference was statistically significant (Cox mixed-effects model: hazard rate=2.45, *z*-value=3.05, *P*=0.002; Fig. 8). Similarly, the odds of returning to the old nest versus not returning was on average three times greater for followers of the robotic leader (straight, sinusoidal and cleaned) than for ants carried manually there (carried and control, Fisher's exact test: MLE odds ratio=3.176, 95% CI=1.031,10.261, *P*=0.037; Table 1).

### Activity of filmed ants compared with carried ants

We found no evidence that tracking with the gantry biased the movement of ants. Potentially, the gantry could be ‘pushing’ the ants because they could be avoiding the camera shadow, which is always on the new nest side of the returning ant (see Materials and Methods, Fig. 2). There was no significant difference between carried and filmed ants in their percentage activity in quadrant 1 and quadrant 4 (GLM: *t*=−0.294, d.f.=29, *P*=0.771; the GLM fitted adequately: Shapiro–Wilk normality test for residuals, *W*=0.974, *P*=0.141).

## DISCUSSION

We have successfully reproduced many of the features of real tandem runs, using a gantry as a robotic leader. Ants that follow this leader to the new nest site, on either a straight or a sinusoidal path, are significantly more successful at finding their way back to the old nest than ones that have been carried and have therefore not learned the route.

There was no significant difference in return success between the ants that had been led on a straight path versus those led on a sinusoidal path. Real tandem runs can be quite convoluted (Basari et al., 2014a; Mcleman et al., 2002; Pratt et al., 2001) but subsequent tandem runs seem often to be straighter than earlier ones (Pratt et al., 2001; Sasaki et al., 2020). The straight paths were, of course, much shorter and more direct than the sinusoidal paths, but the latter may constitute longer lessons and thus may give followers more time to learn their surroundings. It seems possible that in our experiments the different advantages of straight versus sinusoidal paths may have balanced one another out. In addition, it is likely that tandem followers learn both local and more distant landmarks (Basari et al., 2014a; Pratt et al., 2001; Stroeymeyt et al., 2010). In our experiments, we did not employ specific local landmarks because they could have interfered with the movement of the gantry and its accuracy, especially when it was in auto-tracking mode. However, as we have shown that a robotic leader is effective, our work opens up the possibility of new, more detailed studies of the influence of path shapes as well as local and distant landmarks to determine what tandem followers focus their attention on during tandem runs.

Our experimental controls strongly suggest that the moving parts of the gantry were not being used as landmarks by followers and that the shadows cast by the gantry were not influencing the movements of the ants. Moreover, the controls, in which we cleaned the path taken by the robotic leader, show that the successful return paths of the follower ants were not totally dependent on any chemicals deposited along the route. Thus, the experimental results, taken together, strongly suggest that ants that had followed the robotic leader were learning visual landmarks to help them return successfully to the old nest.

We have shown that ants can learn route information from following the robotic leader, despite the leader not laying a trail. When a real ant leads a tandem run, it frequently and repeatedly taps the tip of its gaster onto the ground just in front of the follower (Basari et al., 2014b). Such pheromone trails are not slavishly followed by either returning tandem leaders or followers (Basari et al., 2014a; Sasaki et al., 2020), but may serve as safety lines that eventually mark the general route between a chosen new nest and the old one (Basari et al., 2014a; Pratt et al., 2001).

Typically, tandem followers do not lay trails when they are following a real tandem leader that is laying a trail (Basari et al., 2014b). However, in a preliminary experiment, additional ants to the follower were allowed into the arena as the robotic leader moved towards the new nest closely pursued by a follower. In such runs, we were extremely surprised to observe many ants following the exact (non-cleaned) path of the robotic leader, when it had proceeded both in a sinusoidal and in a straight trajectory (Fig. 5). Indeed, such behaviour seems to resemble mass recruitment via trail pheromones, yet this has never been observed in this species of ant throughout the hundreds of nest choice replicates we have run (Dornhaus et al., 2004; Franks et al., 2003a,b).

There are three ways in which chemicals might be left in the wake of the robotic leader. First, pheromones may drip from the glands on the pin of the robotic leader. Second, traces of pheromones that diffuse into the air from these glands may contact the substrate and adhere to it. Third, the ant immediately following the robotic leader may be laying its own trail. We strongly favour this third possibility for the following reasons. If pheromones had dripped from the pin, these hotspots of attractive pheromones would likely locally arrest the movements of the followers, and this was never observed. Furthermore, such splashes would be few and far between and occur at random along the path, and are therefore unlikely to give rise to the smooth path-following that we observed (Fig. 5). Additionally, the control and carried treatments did not differ significantly in return success or number of crossings of the path of the robotic leader. The second possibility, that some of the volatile pheromones in the wake of the robot might adhere to the substrate, is also unlikely to explain the observations. The laboratory air conditioning and the motion of the gantry itself are likely to have created some turbulence in the air. Thus, it seems unlikely that any airborne pheromones that later adhered to the substrate would have exclusively marked the very precise path that we observed (Fig. 5). This leaves the third possibility: the ant immediately following the pin is laying its own trail. Given that tandem followers often become leaders in due course, their trail laying while following the robotic leader may simply be a case of producing their own safety lines sooner rather than later. This would be a further fascinating example of fail-safe mechanisms in these ants in particular, and social insects and many other organisms in general (Berdahl et al., 2018; Camazine et al., 2001; Franks et al., 2015; Oster and Wilson, 1978; Rajendran et al., 2022; Robinson et al., 2014; Stuttard et al., 2016; White and Diedrichsen, 2010).

Our previous studies of collective decision-making in ants have revealed remarkably sophisticated algorithms that enable them to make swift and accurate decisions, albeit with classic speed–accuracy trade-offs (Franks et al., 2003b), and they can even make rational decisions over nests that fluctuate in quality (Franks et al., 2015). Tandem-running recruitment underpins much of this collective decision-making as the ants use it to build quora (Pratt et al., 2002) so that information is pooled and decisions are corporate. Here, we have successfully constructed a robot to act as a tandem leader. This will facilitate new investigations of individual and collective decision-making in ants.

## Supplementary Material

Supplementary information
