# Supplementary material for: Robotic communication with ants
Source: J Exp Biol. 2022 Aug 9;225(15):jeb244106. doi: 10.1242/jeb.244106 (PMC9440752; doi:10.1242/jeb.244106)
Supplement: Supplementary information [file jexbio-225-244106-s1.pdf]

Table S1. The duration between the time a robot-led ant entered the NN and the time it started its return trip to the ON

ID	Treatment	In NN (min)	In NN (s)	Total (s)	Left NN	Returned to ON	Notes
Out001	straight	15	30	930	y	n	
Out004	straight	14	7	847	y	y	
Out007	straight	5	55	355	y	y	
Out009	straight	4	58	298	y	y	
Out011	straight	3	28	208	y	y	
Out012	straight	11	50	710	y	y	
Out014	straight	24	45	1485	y	n	left and re-entered NN many times
Out016	straight	10	53	653	y	y	
Out018	straight	15	0	900	y	y	left and re-entered NN many times
Out020	straight	1	47	107	y	y	
Out022	straight	13	31	811	y	y	
Out024	straight	4	7	247	y	y	
Out026	straight	21	0	1260	y	y	
Out028	straight	#N/A	#N/A	#N/A	n	n	
Out030	straight	15	30	930	y	y	left and re-entered NN many times
Out032	straight	7	2	422	y	y	
Out002	sinusoidal	1	9	69	y	y	
Out003	sinusoidal	6	36	396	y	y	
Out005	sinusoidal	7	26	446	y	y	
Out008	sinusoidal	11	15	675	y	y	
Out010	sinusoidal	14	30	870	y	y	
Out013	sinusoidal	4	50	290	y	y	
Out015	sinusoidal	21	10	1270	y	y	left and re-entered NN many times
Out017	sinusoidal	2	4	124	y	y	
Out019	sinusoidal	1	58	118	y	y	
Out021	sinusoidal	21	36	1296	y	y	left and re-entered NN many times
Out023	sinusoidal	29	45	1785	y	y	followed beyond 20min to see if lost ant ultimately will find ON
Out025	sinusoidal	7	40	460	y	y	
Out027	sinusoidal	8	22	502	y	y	left and re-entered NN many times

Journal of Experimental Biology • Supplementary information

Out029	sinusoidal	#N/A	#N/A	#N/A	y	n	returned to NN at 19:57min and remained there
Out031	sinusoidal	3	52	232	y	y	
Cleout001	cleaned	#N/A	#N/A	#N/A	y	n	left and re-entered NN many times, entered NN at 21min and remained there
Cleout002	cleaned	14	30	870	y	y	
Cleout003	cleaned	4	38	278	y	y	
Cleout004	cleaned	15	23	923	y	y	
Cleout005	cleaned	13	21	801	y	y	
Cleout006	cleaned	#N/A	#N/A	#N/A	y	n	in NN at 20min
Cleout007	cleaned	3	16	196	y	y	
Cleout008	cleaned	6	35	395	y	y	left and re-entered NN many times
Cleout009	cleaned	#N/A	#N/A	#N/A	y	n	left and re-entered NN many times
Cleout010	cleaned	7	10	430	y	y	
Cleout011	cleaned	6	48	408	y	y	
Cleout012	cleaned	3	45	225	y	y	
Cleout013	cleaned	4	34	274	y	y	
Cleout014	cleaned	#N/A	#N/A	#N/A	y	n	in NN after 20min
Cleout015	cleaned	14	50	890	y	y	left and re-entered NN many times
Cleout016	cleaned	#N/A	#N/A	#N/A	y	n	left and re-entered NN many times, in NN at 20min

Note that OutXXX stands for the outward journey of the same ant as the one with return journey RetXXX (see data file on Dryad).
Similarly, CleoutXXX stands for the outward journey of the same ant as the one with return journey CleretXXX (see data file on Dryad).

Journal of Experimental Biology • Supplementary information
